# Investigation of Using Sky Openness Ratio as Predictor for Navigation Performance in Urban-like Environment to Support PBN in UTM

**DOI:** 10.3390/s22030840

**Published:** 2022-01-22

**Authors:** Chao Deng, Chung-Hung John Wang, Kin Huat Low

**Affiliations:** 1 Air Traffic Management Research Institute, Nanyang Technological University, Singapore 637460, Singapore; dengchao@ntu.edu.sg (C.D.); JohnWang@ntu.edu.sg (C.-H.J.W.); 2 School of Mechanical and Aerospace Engineering, Nanyang Technological University, Singapore 639798, Singapore

**Keywords:** position error, navigation, performance-based navigation (PBN), drones

## Abstract

One of the causes of positioning inaccuracies in the Unmanned Aircraft System (UAS) is navigation error. In urban environment operations, multipaths could be the dominant contributor to navigation errors. This paper presents a study on how the operation environment affects the lateral (horizontal) navigation performance when a self-built UAS is going near different types of urban obstructions in real flight tests. Selected test sites are representative of urban environments, including open carparks, flight paths obstructed by buildings along one or both sides, changing sky access when flying towards corners formed by two buildings or dead ends, and buildings with reflective glass-clad surfaces. The data was analysed to obtain the horizontal position error between Global Positioning System (GPS) position and ground truth derived from Real Time Kinematics (RTK), with considerations for (1) horizontal position uncertainty estimate (EPH) reported by the GPS receiver, (2) no. of visible satellites, and (3) percentage of sky visible (or sky openness ratio, SOR) at various altitudes along the flight paths inside the aforementioned urban environments. The investigation showed that there is no direct correlation between the measured horizontal position error and the reported EPH; thus, the EPH could not be used for the purpose of monitoring navigation performance. The investigation further concluded that there is no universal correlation between the sky openness ratio (SOR) seen by the UAS and the resulting horizontal position error, and a more complex model would need to be considered to translate 3D urban models to expected horizontal navigation uncertainty for the UAS Traffic Management (UTM) airspace.

## 1. Introduction

UAS has gained more popularity over the decades across many industries. As more mission types are explored, UAS are used in many new and creative applications. Since the start of the COVID-19 pandemic when people’s movements were restricted, in order to reduce contact with others, the use of UAS operations in urban environments has been brought to attention [1,2]. For example, Singapore Police Force used drones as a form of contactless patrol [3], and the United Nations International Children’s Emergency Fund (UNICEF) has also acknowledged the usage of drones for delivery and transportation and public space monitoring and guidance in response to COVID-19 [4]. Increasing urban applications of UAS means more operations over populated areas, and these operations should be treated to a high safety level. One of the most important factors contributing to an efficient and safe urban UAS operation would be the navigation performance in Communication, Navigation and Surveillance (CNS). Navigation tools help maintain a continuous precise placement of the UAS to direct or control it. Navigation performance of a UAS mostly depends on Global Navigation Satellite System (GNSS) device on board, which works well in open areas, but the accuracy and reliability of GNSS are often affected by signal blockage and electromagnetic interference [5]. The navigation performance decreases when UAS is in the vicinity of obstructions, which is caused by the reduction in the number of visible satellites and increased multipath effects.

In the Air Traffic Management (ATM) concept, Performance-Based Navigation (PBN) has been introduced to have standardised Required Navigation Performance (RNP) and Area Navigation (RNAV) specifications to facilitate more effective and safe manned aircraft flight operations. Learning from that, a similar concept could be adapted in UAS Traffic Management (UTM) operations, especially in urban environments. For the UAS to be able to operate in a specific category of airspace, the corresponding RNP requirement of that airspace needs to be met by the UAS navigation performance provided by the navigation instruments. This work aims to provide some insight into how the operation environment affects the lateral (horizontal) navigation performance when our self-built UAS is flying close to different types of urban areas.

While GNSS receivers provide real monitoring parameters, such as Horizontal Dilution of Precision (HDOP) and EPH (horizontal covariance), no technology or model exists to predict GNSS performance in urban airspaces short of detailed surveys. An additional experimental setup for hemispheric photography was made at different altitudes to investigate if a correlation exists between GNSS performance and geometric blockage, or the sky openness ratio (SOR), independent from the multipath error in urban airspace. While previous studies have demonstrated a relationship between the visible sky (blockage factor) and positional accuracy, the contribution by the sky openness ratio was not isolated [6]. While no formal correlation had been specified, the building geometries and their blockage of visible sky were included as a parameter in the evaluation of GNSS performance in urban environments, as specified by the European Telecommunications Standards Institute (ETSI) [7]. With the increased availability of a high-quality three-dimensional model for urban planning purposes, a link between the sky openness ratio and GNSS performance could provide a way to predict the latter before the commencement of UTM.

Starting with a background introduction, this paper aims to investigate the correlation between the measured horizontal position error (between GPS and RTK-ground truth), the reported EPH, and the sky openness ratio. Finding a relationship between the first two would allow for real-time navigation performance monitoring in UTM without additional infrastructure, while finding a relationship between the measured error and sky openness ratio could open an avenue for estimating navigation performance that could be expected using the available three-dimensional urban maps. To achieve that, this paper is organised to present the experiment methodology, a short discussion about the result analysis, and followed by concluding remarks.

## 2. Background

Below, this section will discuss the factors affecting navigation performance:

### 2.1. Navigation

Navigation is critically important in any flight mission. Its performance will directly affect the safety of the aircraft, other airspace users, and people and properties on the ground. Global Positioning System (GPS) and Inertial Navigation System (INS) are the most commonly seen UAS navigational equipment onboard. Currently fully operational GNSS include Global Positioning System (GPS), Global Navigation Satellite System (GLONASS), BeiDou Navigation Satellite System (BDS) and Galileo. The basic GPS service provides users with approximately 7.8 m accuracy, 95% of the time, anywhere on or near the surface of the earth [8]. However, in certain operation conditions, the GPS accuracy would be degraded to a level that could not ensure safe flight. For the safety of the community on the ground, UAS operating in urban environments requires navigation with high accuracy, especially for missions, such as infrastructure inspection, area survey and last-mile delivery that requires flight between narrow urban canyons [9,10] or very close to a high density of human structures. Therefore, additional navaids (Visual Inertial Odometry, Beacon, Wi-Fi, Radar, etc. [11,12,13]) would be needed to improve the navigation performance for UAS operating in those challenging areas and when the achievable navigation performance is not good enough to allow UAS to operate safely. Therefore, to ensure safe UAS operations, based on the PBN concept in ATM, there is a need to understand the achievable navigation performance for different airspaces in different operation environments.

### 2.2. Required Navigation Performance (RNP) in ATM and Its Adaptation in UTM

RNP in ATM is a type of PBN used in the current aviation industry that requires a clear understanding of the CNS performance involved in the operation to help aircraft follow specific flight paths with high accuracy. Aircraft would need to fulfil the specific level of performance required in order to operate in that particular airspace. For example, RNP 1 means the aircraft navigation should be able to calculate its position with a radius of 1 NM. The performance requirements could be considered in 5 main indicators: accuracy, integrity, continuity, availability and functionality in the ICAO PBN Manual [14]. In addition, On Board Performance Monitoring and Alerting (OBPMA) is required in RNP to ensure the required CNS performance is maintained throughout the operation by alerting Air Traffic Control (ATC) if the performance requirement for the approved operation could no longer be met [15].

Like aircraft operations in ATM, UAS in UTM operation also relies heavily on CNS technologies. Recognising the future trend of incorporating UAS activity into the current airspace system [16], the ATM PBN concept could be adopted in UTM to allow UAS to operate more accurately, safely and efficiently. However, since the operating environment of UAS is distinctive from manned aircrafts, the UAS navigation performance requirements would also need to be redefined. One of the characteristic features of UAS operation in urban airspace is that there are forests of high-rise buildings and complex structures. UAS flying in low-altitude airspace would require high accuracy navigation capability to keep themselves a safe separation distance from any terrain or construction. By categorising the navigational performance requirement at specific operation conditions, pilots may safely be confident if he/she is suitably prepared to approach that airspace.

### 2.3. Navigation Error

Navigational error is the key to be determined before defining navigational performance requirements. Positioning accuracy not only affects navigation performance, but also has a consequence on other UTM areas, such as airspace capacity and path planning [17,18], etc. Path Definition Error (PDE), Navigation System Error (NSE) and Flight Technical Error (FTE) are the three main component errors in the total system errors (TSE) in PBN. NSE is the subject of this experiment. This could be the limiting factor to the application of RNP and is commonly caused by GNSS inaccuracy in urban environments [19]. NSE could be derived by finding the difference between the true position and its displayed position of a UAS.

The Real Time Kinematics (RTK) [20] method could be applied to find the UAS true location. It consists of a stationary reference receiver, the base station, for the background navigational uncertainty, and a rover installed on the UAS as the moving receiver. Many papers have proven the RTK system’s high accuracy, which could reach as low as cm-level. It was found in Ref. [21] that RTK could measure a horizontal accuracy of within 1 cm + 0.5 ppm. At a height of 100 ft, the P4 RTK has a relative vertical accuracy of 2 cm and a horizontal accuracy of 1.2 cm [22]. In ref. [23], the horizontal accuracy of RTK was found to be 14 mm ± 4 mm.

### 2.4. Signal Blockage

In urban areas near obstructions, other than multipath effects [24] and electromagnetic interference [25] that can cause additional error in NSE, signal blockage is also one of the factors that hinder navigation performance. The signal transmitted from the satellite is so weak that any blockage would severely affect its accuracy and signal availability. As the UAS goes nearer to obstructions, its area of sky that could see satellites would be less. It was found in ref. [26] that as the height of the buildings or the elevation angle increases, the number of visible satellites reduces. Similarly, low-altitude operations could bring on problems of sky blockage, and the GNSS performance would be severely attenuated with a reduced number of visible satellites. Under these conditions, relatively poor visible satellite geometry would be formed, and large Dilution of Precision (DOP) values would be obtained and eventually lower position accuracy. In ref. [27], as the cut-off elevation angle reduces from 15 to 5, the DOP value is reduced to 2.6. Therefore, the height of the surrounding buildings above the UAS would be the main limiting factor of GNSS visibility, which determines the minimum elevation of a visible satellite, or the elevation mask [28].

It was tested using Single Point Positioning (SPP) with the simultaneous observations of the GPS/GLONASS/BeiDou systems showing that at an elevation mask angle of 10°, the accuracy was improved by 10%; however, at an elevation mask angle of 30°, the number of available satellites are less than 4 at most of the times, which is not enough to obtain a solution [29]. Ref. [30] even researched the effect on GPS navigation due to the blockage of GPS signals by the international space station (ISS). It was found that there is a need to aid GPS when its distance to the ISS is less than 60 m because below 60 m the GPS satellite visible is less than 4. When it is less than 10m, the GPS outage duration is 99.99% [31]. In Land-Vehicle Navigation (LVN), GPS signal blockage is also a major problem. Even though high elevation mask angle negatively affects navigation performance, it was found that with the use of GPS/GLONASS/GALILEO using Precise Point Positioning (PPP), it was able to have high navigation performance with elevation angles as high as 40° [32].

Similarly, previous flight tests have shown some sensitivity of measured horizontal error to changes in flight altitude, though the contribution by geometry and sky blockage has yet to be established [33]. The sky blockage ratio could be estimated by using a similar concept in forest canopy cover estimation [34,35,36,37] by using hemispherical photography of the operating environment taken by a 180° fisheye camera.

## 3. Design of Experiments and Experiment Setup

**Figure 1:**
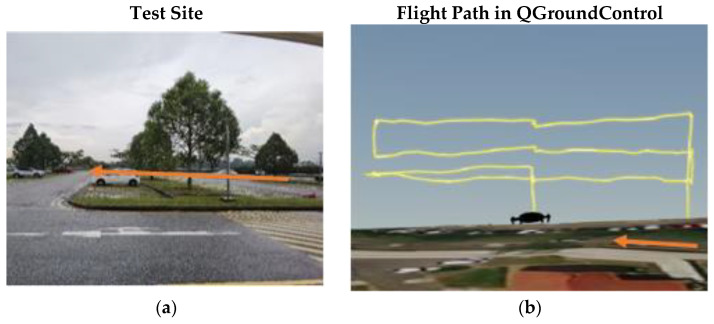
(**a**) Carpark F, NTU. (**b**) Flight Path at Carpark F.

**Figure 2:**
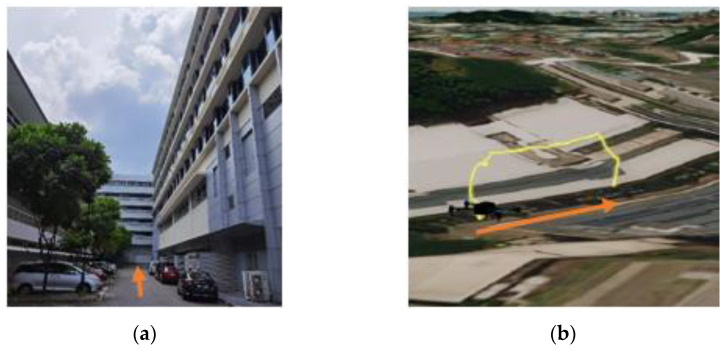
(**a**) Carpark N3.1, NTU. (**b**) Flight Path at Carpark N3.1.

**Figure 3:**
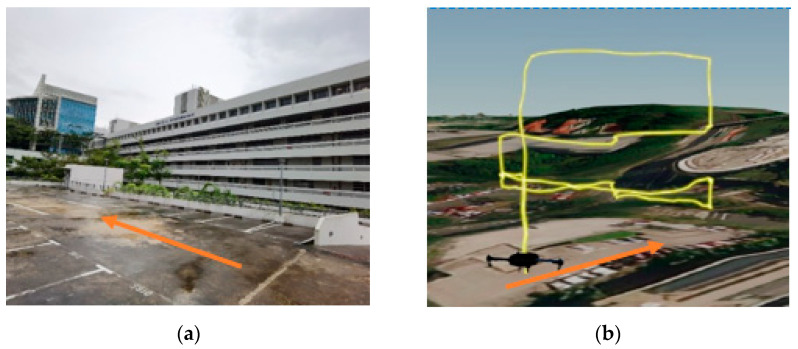
(**a**) Carpark N2.1, NTU. (**b**) Flight Path at Carpark N2.1.

**Figure 4:**
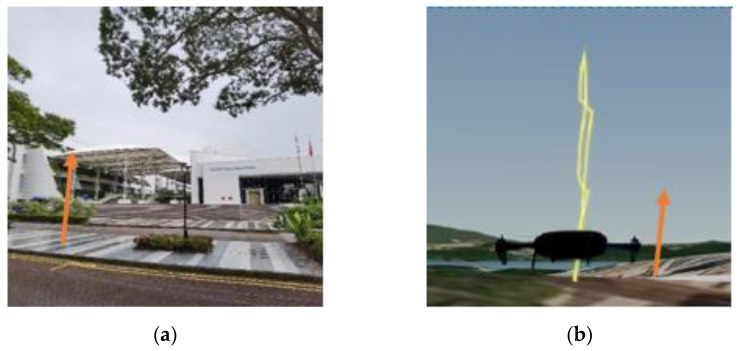
(**a**) Outside Admin Building, NTU (**b**) Hover Path at Admin Building.

The research has been conducted at the Nanyang Technological University (NTU) campus, located on the west side of Singapore. The aim of the experiments is to understand drone lateral navigation performance in terms of accuracy/error when the sky openness ratio changes with the drone’s movement between obstructions of different urban conditions. Four test sites have been identified within the campus that are representatives of the urban environment, as listed below with the orange arrows in each set of pictures, indicating the same alignment direction:

(a) Carpark F, Figure 1a: Open space sparsely covered with short (<10 m in height) trees.
(b) N3.1 Carpark, Figure 2a: Entering from an open carpark into a narrow urban canyon with 3 sides blocked by buildings.
(c) N2.1 Carpark, Figure 3a: Half-canyon with an L-shape building along the South and East sides of the flight area.
(d) Admin Building, Figure 4a: Building clad with reflective surface and flanked by tall trees (~15 m) in the vicinity of the hover location.

The flight locations and paths were selected to transit through a wide range of sky openness ratios commonly seen in urban areas. This includes wide-open space common in city park areas and plazas; urban canyon with various building coverage and orientations relative to the flight path often seen in central business districts; single large building flanked by tree coverage commonly seen in Singapore’s science and industrial parks, where tall tropical trees along the roads are the norm.

**Figure 5:**
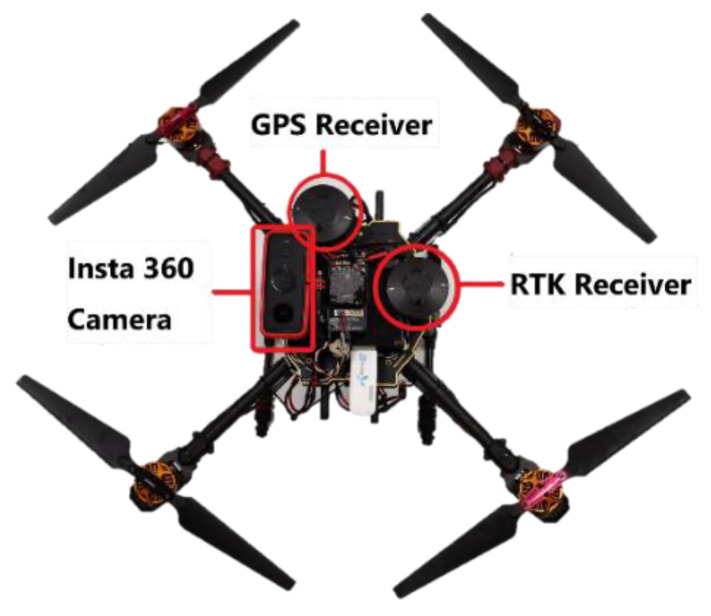
Tarot Ironman 650 with GPS Receiver, RTK Receiver and Insta 360 Camera Facing Upwards.

**Figure 6:**
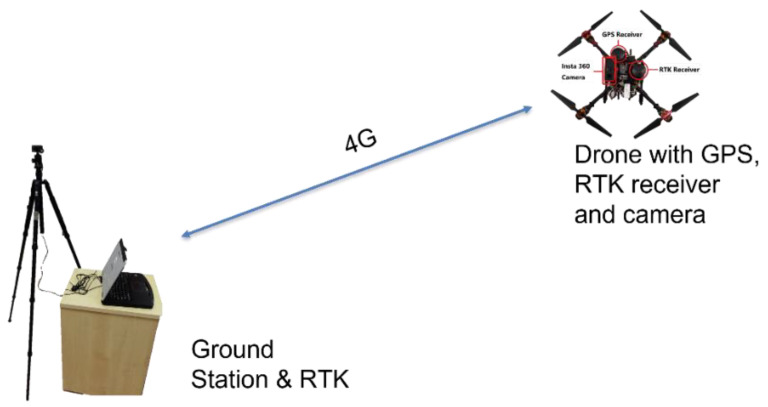
Experiment Setup.

A self-assembled drone (~2.6 kg) shown in Figure 5 built using the Tarot Ironman 650 kit with Pixhawk 2.1, has been used in the flight tests. There is an Intel^®^ NUC Kit with 7th Generation Intel^®^ Core™ Processors installed to transfer telemetry via 4G Figure 6. There is also an Insta360 ONE X2 camera on the drone facing upwards to take photos when the drone is flying at different photo points. The UAS is configured to capture the positional data with a commercially available Here GPS receiver and a Here+ RTK GPS receiver onboard.

**Figure 7:**
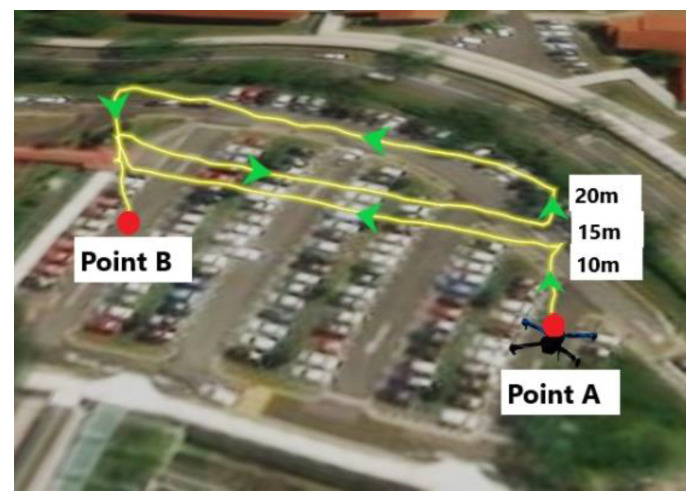
An Illustration of the Flight Path and Points A & B in Car Park F.

**Figure 8:**
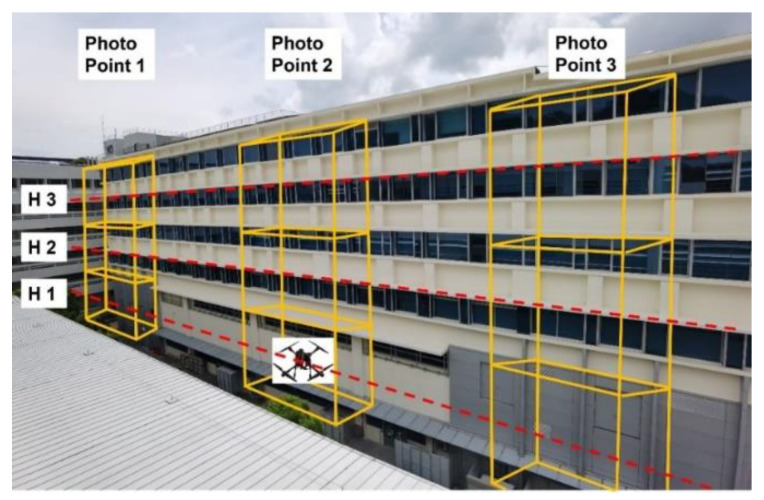
Illustration of Different Points at Three Altitudes for the N3.1 Location.

The mission was designed to fly at different heights at 10 m, 15 m and 20 m above ground level (AGL) and at a speed of 1m/s in straight lines. As illustrated in Figure 7, at open-space Carpark F, the flight path is firstly from point A to point B at 10 m AGL, elevated to 15 m at point B and flying back to point A, while maintaining the altitude, then elevated to 20 m and flying to point B again at the same height. The flight path at Carpark F was to fly along the latitudinal direction; similar flight paths were conducted at N3.1 (along latitudinal direction) and N2.1 (along longitudinal direction). Since there were tall trees in the surroundings, the drone was manually controlled and hovered at the 3 altitudes levels, 10 m, 15 m and 20 m above ground level (AGL), at the Admin Building; only one set of complete flight tests were performed at this location. Since NSE will be our focus, flights at Carpark F and N2.1 were conducted in mission mode by QGroundControl [38] to minimise FTE due to operator inputs from occurring, while flights at N3.1 and Admin Building were conducted manually due to safety concerns limited by the operating environment (proximity to tall building) and poor navigation reception (N3.1). Photos will be taken at 3 photo points at each altitude, as illustrated in Figure 8. The photo points are the 2 ends and the middle of the flight paths. For flights at the Admin Building, there will be 3 photos, 1 at each altitude.

The GPS will be the main receiver to navigate the drone during operation and the RTK receiver will be providing the information for comparison for post-flight analysis. Furthermore, the flight tests will be conducted during weekends to avoid peak carpark use hours for safety reasons. After the flight, the flight log was extracted from the system and analysis will be conducted.

The data collected will be analysed with considerations for:

i. No. of visible satellites vs. flight altitude.
ii. Horizontal position errors by comparing reported position and actual position (NSE).
iii. Sky openness ratio vs. horizontal position accuracy.

## 4. Results and Discussion

After the flight data were collected from the 4 test sites, graphs were plotted for analysis with considerations for number of satellites, horizontal position error (EPH) and measured horizontal position error, and sky openness. The RTK positions were used as ground truth in all cases with the RTK base station setup a few hours before the flight to obtain a survey-in position fix with accuracy <1 m.

### 4.1. Number of Visible Satellites vs. Flight Altitude

**Figure 9:**
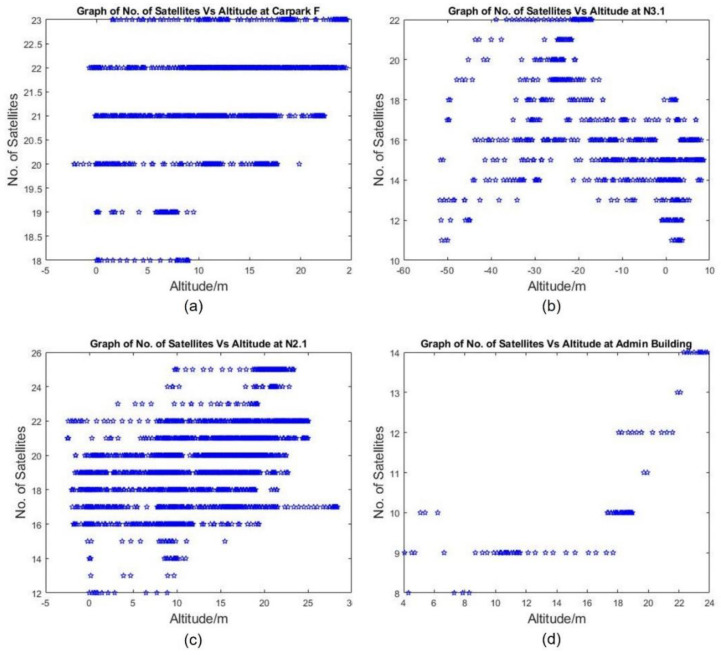
Graphs of No. of Satellites vs. Altitude at the 4 Locations: (**a**) Car Park F, (**b**) N3.1, (**c**) N2.1 and (**d**) Admin Building.

At carpark F, the number of satellites used by the GNSS receiver ranges from 18 to 23 (Figure 9a), while a minimal of 4 satellites are needed to obtain a position by GPS. However, as suggested in the rest of the Figure 9 sub-plots, the number of viewable satellites is not a good indicator of GNSS performance. Note that the number of viewable satellites was compared at different reported altitudes due to the data collection missions being performed in mission mode (apart from the flights at N3.1 and the Admin Building, which were flown manually), i.e., the UAS was guided by the fused GPS altitude. The fused altitudes utilise the barometric altitude relative to take-off altitude (GPS with reference to WGS84 ellipsoid) with the raw altitude output from the GPS receiver. It is used for navigation due to the large uncertainty associated with raw GPS altitude.

The number of satellites in view at different raw GPS altitudes were further collected at N3.1, N2.1, and the Admin Building and shown in Figure 9b–d, respectively. Note that even though there were enough satellites visible for positioning, the raw GPS altitude shows large variability, e.g., at N3.1 (narrow urban canyon), the drone was reported at as low as −50 m, rendering Figure 9b unreliable. Additionally, several data points in Car Park F (open field) show a reported altitude below that of ground level even though the take-off and landing were performed at the same location and the openness of the test site would allow the GPS to operate with the best accuracy. In general, however, the observation was that as the drone’s altitude increases, a larger number of satellites could be used.

### 4.2. Measured Horizontal Position Errors

**Figure 10:**
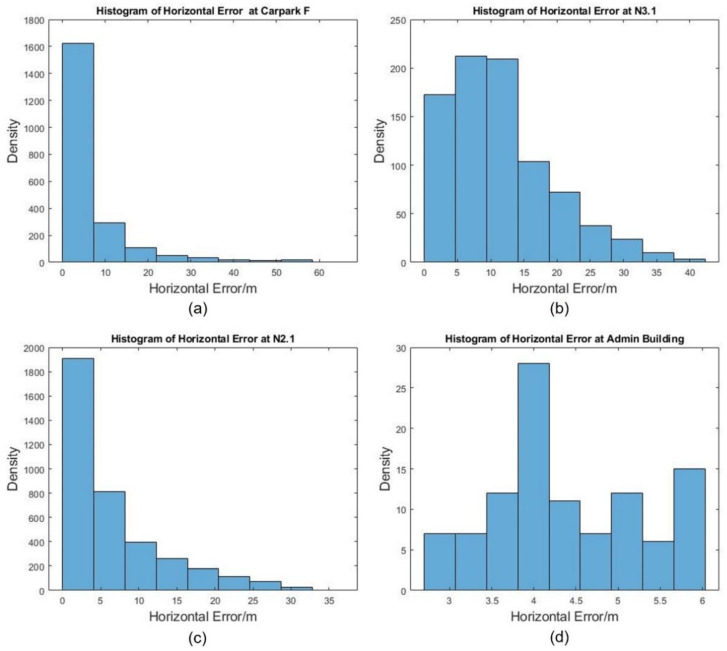
Histograms of Horizontal Position Errors at the 4 Locations: (**a**) Car Park F, (**b**) N3.1, (**c**) N2.1 and (**d**) the Admin Building.

Measured horizontal position errors were derived by finding the difference between GPS and RTK (used as ground truth) reporting positions at the same timestamp. Figure 10a–d shows the histograms of measured absolute horizontal position errors at the 4 locations, respectively. For locations at F, N3.1 and N2.1, the horizontal errors are generally normally distributed around ±0 m. For the Admin Building, no distinct trend was observed. This might be because the Admin Building is clad with reflective surfaces with tall trees around the flight path, thus introducing additional uncertainties via signal reflection (by the cladding) and signal diffusion (by the tree branches and leaves), though further testing would be needed to confirm this hypothesis.

### 4.3. Horizontal Position Error (EPH) vs. Measured Horizontal Error

EPH is one of the outputs provided directly by the GPS receiver; it is a real-time estimation of GPS horizontal position accuracy using its covariance accounting for signal availability, strength, dilution of precision, and pseudo-range residuals [39]. Hence, it could contribute as a metric in UAS trajectory conformance monitoring. Since the EPH is obtained directly from the GPS receiver, it could be affected by the same factors influencing GPS position accuracy. The reported EPH values are compared to the measured horizontal error, which is obtained by taking the difference between the reported GPS position and the reported RTK position. The RTK position is used as the ground truth, as the differential correction transmitted by its base station would enable position measurements with centimetre accuracy.

**Figure 11:**
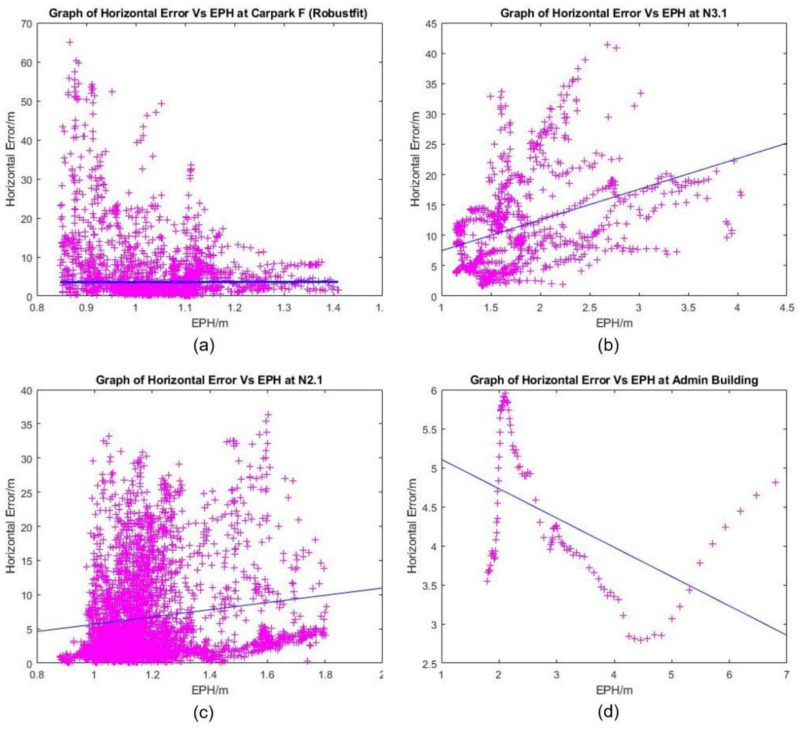
Graph of Measured Horizontal Error vs. EPH with Least Square Line in Blue at the 4 Locations: (**a**) Car Park F, (**b**) N3.1, (**c**) N2.1 and (**d**) the Admin Building.

Graphs of EPH vs. measured horizontal errors with least square line in blue at various locations are presented in Figure 11. Measured horizontal error and EPH are expected to be positively correlated in this simple trend analysis, and this is reflected in data collected at N3.1 and N2.1. On the other hand, Carpark F data showed several outliers with large deviations, though the use of robust fit removed most of their influence. In UAS flight control, the raw GPS outputs were smoothed using the extended Karman filter (EKF) to minimise the impact of these outliers on flight handling. Note that only a small number of flight data collected at the Admin Building were deemed valid due to hardware issues during the flight, and the negative correlation between measured horizontal error and EPH that was observed might not be statistically significant.

### 4.4. Visible Sky vs. Horizontal Positioning Error

**Figure 12:**
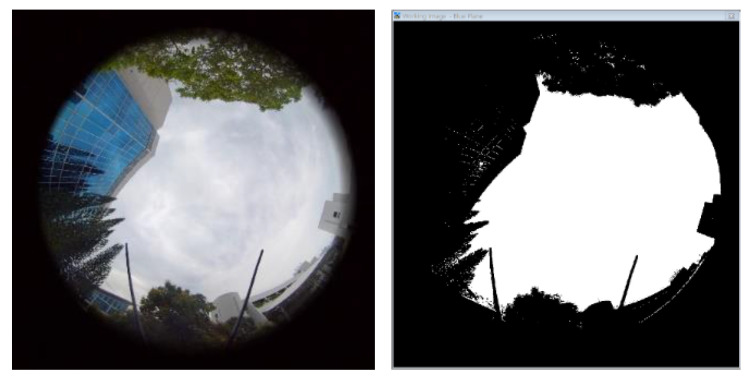
(**Left**) Photo before Process; (**Right**) Photo after applying Blue Filter and Threshold Value.

As illustrated in Figure 5, photos were taken using the Insta360 camera during flight to measure the sky openness ratio while the drones fly past 3 photo points at each altitude level. Only the images from the upward facing camera were used to avoid interpolation artefacts from image stitching. Therefore, the UAS would need to remain hovering in place during the photo-taking process to minimise the impact of vehicle tilt. The photo points are located at the two ends of the flight paths and the middle point of the path. Gap Light Analyzer (GLA) [40] software was used to calculate the percentage of openness of the photo taken. While analysing sky openness, the GLA software was set to have a blue filter and threshold values between 170 to 223. The filter threshold was adjusted manually so that each pixel was classified as either a sky (white) or non-sky (black) as accurately as possible. Picture below (Figure 12) is an example of after applying blue filter and threshold value of 190. The dark clouds are filtered off to avoid ambiguity with the classification of sky or non-sky. Even though the image classification process works well in most cases, sometimes no single threshold value could be used to separate sky and non-sky regions. Thus, there would be some uncertainties associated with the sky openness ratio.

**Figure 13:**
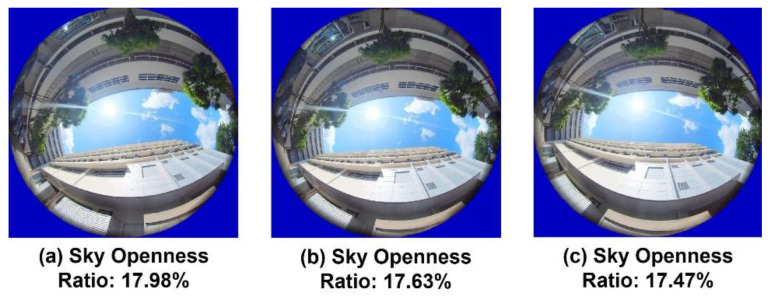
Hemispheric Photo Taken at 1 m Apart near N3.1 to Illustrate the (Lack of) Sensitivity of Sky Openness Ratio to Small Variation in Photo Position Relative to the Designated Photo Points.

As the photo exports and position logs are only available in 1 s intervals, interpolations are needed to match the EPH and horizontal uncertainty with the location at which the photo was taken. Therefore, the sensitivity of the sky openness ratio to minor variation in position would need to be investigated; an additional photography session was conducted at ground level (~2 m AGL) near N3.1 (Figure 13) with a distance interval of 1 m along the road divider line, and the resulting sky openness ratios from Figure 13a–c are 17.98%, 17.63% and 17.47%, respectively. Therefore, it could be assumed that linear interpolation of location coordinates using nearby data at ±1m (or one flight log interval) away would not result in significant altercation to the correlation between horizontal position error and sky ratio for that photo point.

#### 4.4.1. Results from Carpark F

As illustrated in Figure 1, the Carpark F test site is an open area at the top of a hill with very few buildings and trees around it. It is no surprise that the sky ratio obtained here is the highest of the four sites. Furthermore, due to its openness, the variation in sky ration at various Photo Points and altitudes is small. The EPH and measured horizontal error data near the Photo Points were extracted by matching the reported RTK latitude in the flight log.

**Figure 14:**
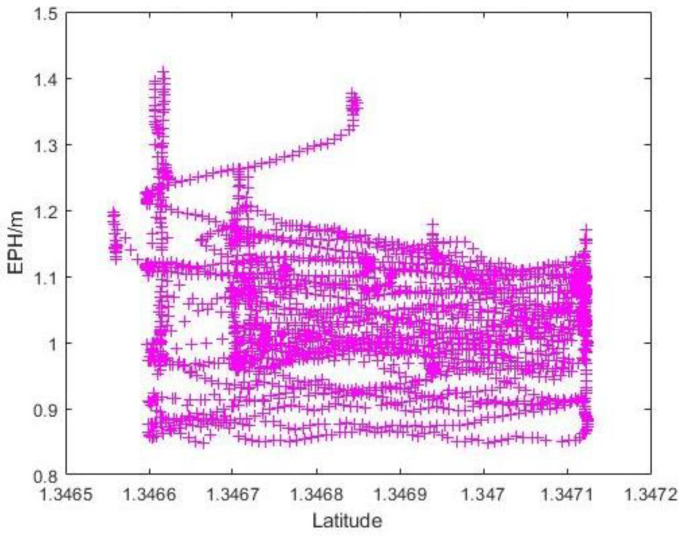
Graph of EPH vs. Latitudinal Value at Carpark F.

**Figure 15:**
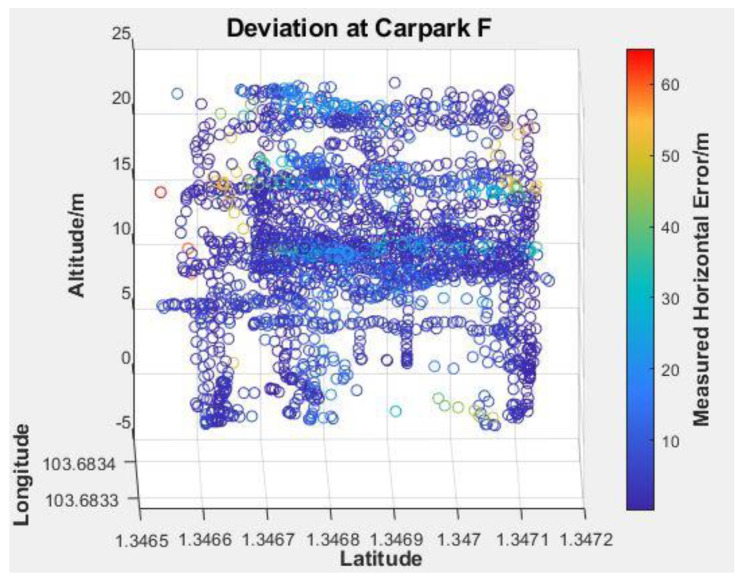
Position Distribution 3D Scatter Plot of Reported GPS Positions with Marker Colour Showing Markers Coloured with Measured Horizontal Error.

**Figure 16:**
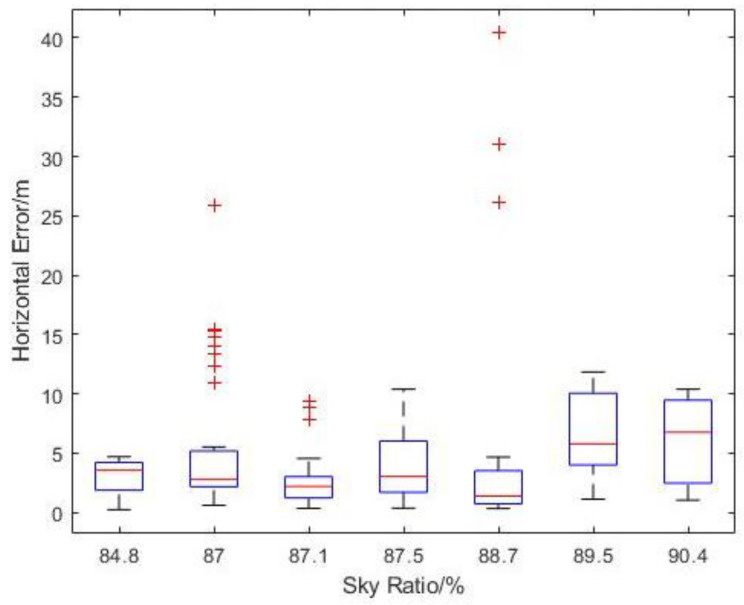
Boxplot of Measured Horizontal Error vs. Sky Ratio at Carpark F.

Since the flight missions were performed along the latitudinal direction at Carpark F, the lower-latitude end of Figure 14 would correspond to Photo Point 1 in Table 1. As expected, the variation in reported EPH remains small (<1 m) throughout the flight with no discernible trend. To better understand the existence of large outliers shown in Figure 11a, a 3D plot of reported GPS positions with markers coloured with measured horizontal error is generated as Figure 15. This identifies the largest horizontal error being near either end of the flight routes where yawing takes place and suggests some form of instrument interference might have taken place that necessitate the re-establishing of GPS position after the yawing manoeuvre. The impact of sensor interference due to change in UAS orientation could be mitigated by removing the flight log entries associated with the end-of-route manoeuvre from the analysis. Similar to the observations in Figure 14, the measured horizontal error over the range of sky openness ratio observed in Figure 16 showed small variation once the outliers were removed.

#### 4.4.2. Results from Building N3.1

Compared to the open field at Carpark F, the test site at N3.1 is much more restricted, with six-story buildings on two sides of the flight route (along the latitudinal direction) and another similarly tall building at the endpoint of the track (Photo Point 3, with the lowest latitude value). Due to the close proximity to buildings, with the corridor slightly greater than 15 m at the narrowest point, and the difficulty with obtaining GPS position fixes, the missions were hand-flown by qualified Singapore unmanned aircraft pilot licence (UAPL) holders.

**Figure 17:**
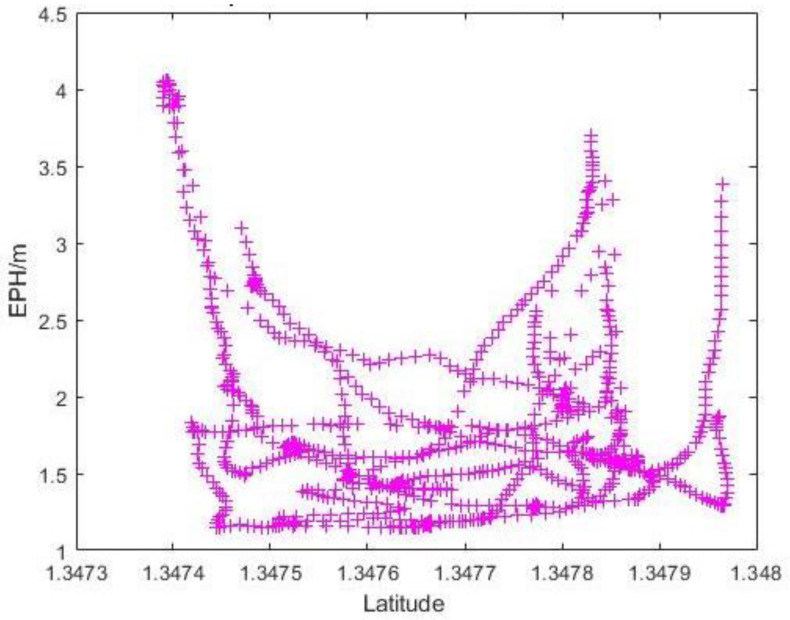
Graph of EPH vs. Latitudinal Value at N3.1.

The sky openness ratio decreases as the UAS travels into the urban canyon, as seen in Table 2. Interestingly, the reported EPH remains low throughout the flight and does not show the expected upward trend as the UAS travels into the lower sky ratio area (Figure 17 Graph of EPH vs. Latitudinal Value at N3.1). This would indicate that the variation in the reported GPS horizontal position is small, even though the horizontal dilution of precision would be high. This could be due to a filter that removed all data points that failed to achieve 3D fix from the data set.

**Figure 18:**
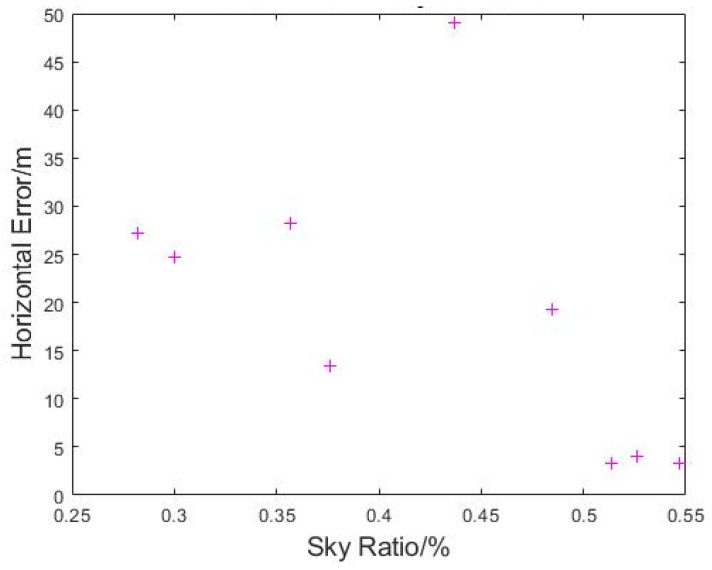
Plot of Measured Horizontal Error vs. Sky Ratio at N3.1.

Due to difficulties with obtaining GPS position fix during this flight, much of the flight data were not usable for the measured horizontal error analysis. The performance of test flight using manual control also complicated the process of matching the measured horizontal error data to specific sky ratios. Instead of relying on RTK data to perform the match, the timestamp from the video from take-off time is matched with the timestamp of position data output. The mismatch between the intervals from photo-taking and data output were resolved using linear interpolation with the results shown in Figure 18. While a general trend of decreasing horizontal error (from 27 m to 3 m) with an increase in sky ratio (from 28% to 55%) could be seen, the number of usable data points is not sufficient to draw a statistically valid conclusion.

#### 4.4.3. Results from Building N2.1

The test site at N2.1 is located on the top deck (third level) of a multistorey carpark with a 6-story tall building on the South side parallel to the flight track and another 6-story tall building near the end point (East side) of the flight track. Note that the hilly nature of the NTU campus meant that the ground floors of the buildings were not lined up.

**Figure 19:**
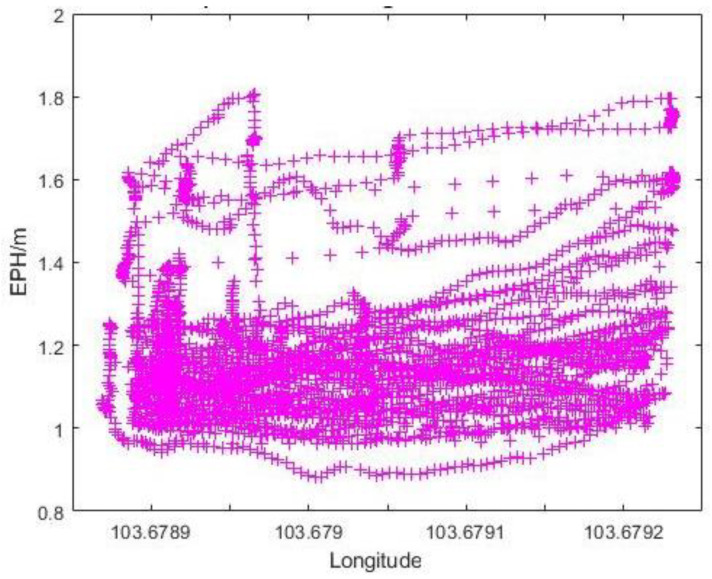
Graph of EPH vs. Longitudinal Value at N2.1.

The missions were performed Eastwards along the latitude line, i.e., Photo Point 1 is at the lowest longitude number and Photo Point 3 at the highest. The EPH showed a slight upward trend as the UAS moves along the planned path (Figure 19), which is associated with a reduction in the sky openness ratio (Table 3). Larger variations in EPH at the same longitude would be likely due to differences in altitude, a factor that should be accounted for in the sky ratio calculation.

**Figure 20:**
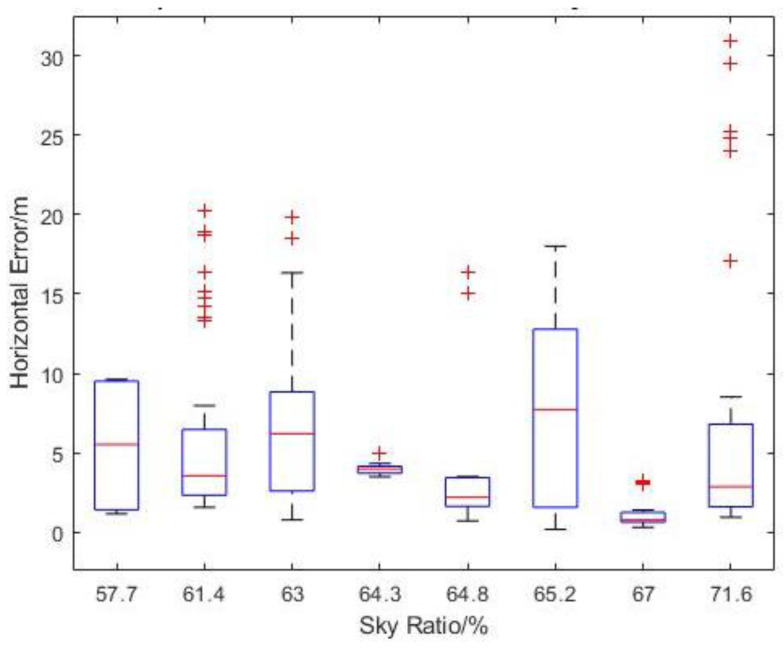
Boxplot of Measured Horizontal Error vs. Sky Ratio at N2.1.

Similarly, the measured horizontal error showed a slight reduction in mean values with the increase in sky openness ratio after the outliers are removed from the statistic (Figure 20). There does not appear to be a consistent standard deviation for the measured horizontal error values across the different sky ratios. One interesting observation is that both the highest mean horizontal error and largest outlier error occurred at the highest flight level of 20 m AGL, which is the opposite of the expectation, as higher altitude should have resulted in less obstructions and interferences to the GNSS signal.

#### 4.4.4. Results from the Admin Building

The Admin Building test site is located on a road divider flanked by a six-story tall glass-clad building at 10 m to the South side, a two-story lecture theatre at 30 m away in the North, and large Rain Trees 5 m to the East and West. Due to the proximity to trees, the missions were flown manually with data collection only along the vertical direction to reduce collision risk.

**Figure 21:**
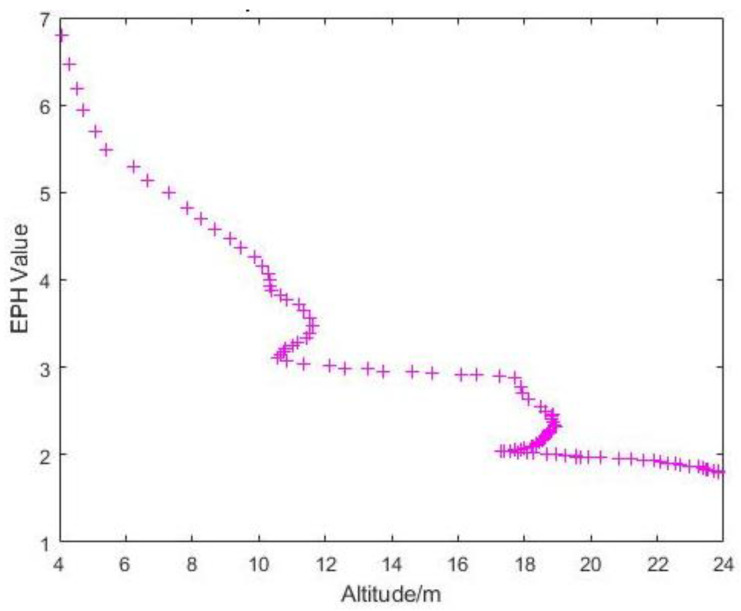
Graph of EPH vs. Altitude at the Admin Building.

**Figure 22:**
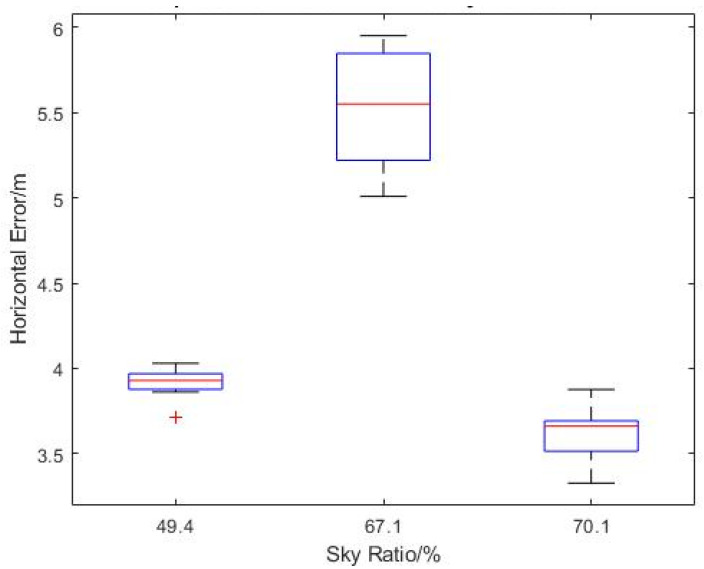
Boxplot of Measured Horizontal Error versus Sky Ratio at the Admin Building.

For the flights in the Admin Building, since it was in a hovering state, change in altitude was the main variable. As expected, the sky ratio increased with the increase in altitude (Table 4). From Figure 21, it can be seen that the maximum EPH was observed at the lowest altitude (most probably because of blockage by obstacles like trees) and decreases as altitude increases, which correspond to an increase in the sky openness ratio. However, in Figure 22, the measured horizontal error does not follow a similar trend. The average measured horizontal error actually increased from ~4 m to ~5.6 m with the increase in sky ratio from 49.4% to 67.1%, before reducing to ~3.6 m at a sky openness ratio of 79.1%. This could suggest that some form of GNSS interference was experienced at 15 m AGL, while ruling out a contribution from direct signal blockage by buildings and diffusion by foliage. It should be noted that the loss of GPS accuracy was not observed in the EPH plot for the same flight (Figure 21), indicating that the source of this inaccuracy does not contribute significantly to the horizontal covariant estimation. Further measurements at that location would be needed to identify the source of this increase in horizontal error.

## 5. Conclusions

The initial observation by analysing the flying test results obtained showed that the number of satellites visible to the UAS increases as the mission altitude increases in an urban-like environment. A preliminary analysis of the measured horizontal error reported by the GPS receiver also showed a similar trend of increasing accuracy with increasing altitude [33]. This observation led to the hypothesis that the physical line-of-sight blockage of the GNSS signal is the dominant factor affecting horizontal accuracy in urban environments. If true, this would imply that the GNSS-based navigation performance in the entire urban airspace could be estimated for use in UAS traffic management, thus enabling navigation performance differentiated traffic flow. To accomplish this, the determination of actual horizontal error between the GPS reported position and some ground-truth reference at various locations and altitudes was needed. Additionally, the use of horizontal covariance (EPH) reported by the GPS receiver as an indicator for navigation performance monitoring for PBN conformance was also investigated.

The measurement of horizontal position error using GNSS navigation was performed by collecting GPS and RTK data at four different locations: Carpark F, N2.1, N3.2 and the Admin Building on the NTU campus. Measurements were taken at each location at three different altitudes: 10 m, 15 m and 20 m above ground level. The data was collected using the Here GPS and Here+ RTK receiver installed on a self-built drone with an additional Insta360 camera installed to collect hemispherical photos for sky openness measurement. The latter was to address the hypothesis that line-of-sight blockage of the GNSS signal by obstacles is the dominating factor affecting the horizontal accuracy of positions reported by GPS receivers. The measured horizontal position errors were calculated by finding the difference between GPS and RTK coordinates at the same timestamp. The test flights were performed with mission mode whenever possible to reduce external influences, such as electro-magnetic interference by the controller input or GNSS signal blockage due to large changes to UAS attitude.

In general, the EPH values reported by the GPS receiver have been shown not to be a good predictor for the actual horizontal error experienced by the UAS. This was shown with the least square analysis in Figure 11, where a negative correlation has been shown between EPH and measured horizontal error at some of the test sites. Furthermore, the reported EPH showed consistently low variability even in challenging environments, such as the N3.1 test site. While it would be impossible to rule out firmware-specific issues with EPH computation without comparison with other GPS receivers, the currently available EPH is clearly not suitable for GNSS performance monitoring to support PBN in UTM.

The hemispherical photo taken with the upper fisheye lens of the Insta360 were used to evaluate the ratio of sky, hence the proportion of GNSS satellites that are visible to the GPS receiver (the sky ratio). A larger sky visible ratio would suggest a lower dilution of precision from satellite geometry. While a general trend of a decrease in measured horizontal error with increasing sky ratio could be seen, the effect does not display great sensitivity to changes in the sky ratio; in some cases, a negative correlation between measured horizontal error and sky ratio could be observed. This would suggest that changes in dilution of precision are not the dominant contributor to changes in position error. The flight test results also suggest that the measured horizontal error does not directly correlate to the flight altitude; thus, the single altitude (e.g., road-level) measurement of GNSS performance might not be sufficient to predict the navigation performance for the UTM airspace.

Overall, the test results showed that the usage of sensor-supplied EPH values would not be sufficient for navigation performance monitoring. It also showed that a simplified model using line-of-sight blockage of the GNSS signal is not a good predictor of changes in horizonal position uncertainty that could be expected in the UTM airspace. Possible future works that could help address this shortcoming could include the incorporation of a more complex GNSS performance model, e.g., multipath model or expected satellite positions, for verification with experimental data; the modelling of GNSS signal with detailed mapping or continuous sky ratio measurement for the flight area could also help support multi-variable analysis to identify factors that led to the unexpected negative correlation between measured horizontal error and sky ratio. The goal will be to identify a sufficiently simple and generalisable model for navigation performance through different UTM flight levels to forego the need to survey the entire UTM airspace before implementing the PBN concept or navigation performance differentiated routing/traffic management. Future works can also extend the current study to urban-like environments [41].

## Figures and Tables

**Table 1:** Sky Ratio at Carpark F.

Carpark F	Photo Point 1	Photo Point 2	Photo Point 3
20 m AGL	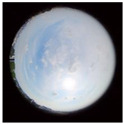 88.9%	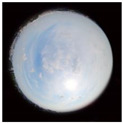 89.5%	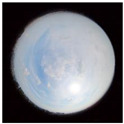 90.4%
15 m AGL	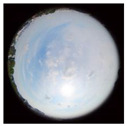 87.1%	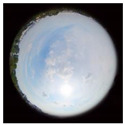 87.5%	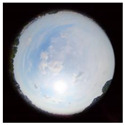 88.7%
10 m AGL	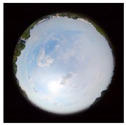 85.7%	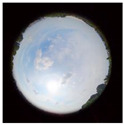 87.0%	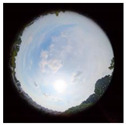 84.8%

**Table 2:** Sky Ratio at N3.1.

N3.1	Photo Point 1	Photo Point 2	Photo Point 3
20 m AGL	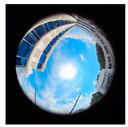 54.7%	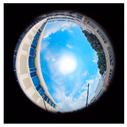 52.6%	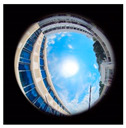 43.7%
15 m AGL	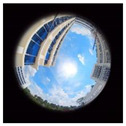 51.4%	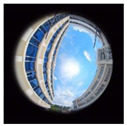 37.6%	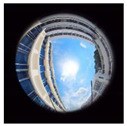 35.7%
10 m AGL	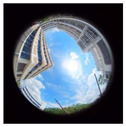 48.5%	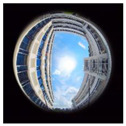 30.0%	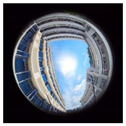 28.2%

**Table 3:** Sky Ratio at N2.1.

N2.1	Photo Point 1	Photo Point 2	Photo Point 3
20 m AGL	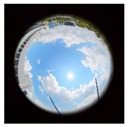 71.6%	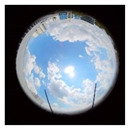 65.2%	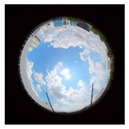 64.8%
15 m AGL	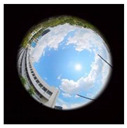 67.0%	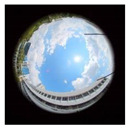 63.0%	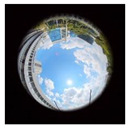 58.1%
10 m AGL	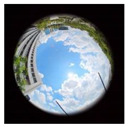 64.3%	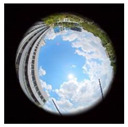 61.4%	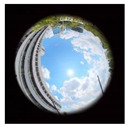 57.7%

**Table 4:** Sky Ratio at the Admin Building.

Admin Building	Hover at Photo Point
20 m AGL	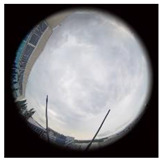 70.1%
15 m AGL	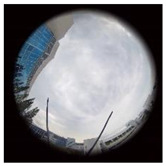 67.1%
10 m AGL	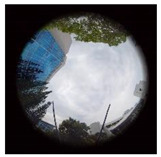 49.4%

## Data Availability

Not applicable.
